# Breathing is coupled with voluntary action and the cortical readiness potential

**DOI:** 10.1038/s41467-019-13967-9

**Published:** 2020-02-06

**Authors:** Hyeong-Dong Park, Coline Barnoud, Henri Trang, Oliver A. Kannape, Karl Schaller, Olaf Blanke

**Affiliations:** 1 Laboratory of Cognitive Neuroscience, Center for Neuroprosthetics and Brain Mind Institute, Swiss Federal Institute of Technology (EPFL), Geneva, Switzerland; 2 Department of Clinical Neurosciences, Division of Neurosurgery, Geneva University Hospitals, Geneva, Switzerland; 3 Faculty of Medicine, University of Geneva, Geneva, Switzerland; 4 Department of Neurology, University of Geneva, Geneva, Switzerland

**Keywords:** Cognitive neuroscience, Sensorimotor processing

## Abstract

Voluntary action is a fundamental element of self-consciousness. The readiness potential (RP), a slow drift of neural activity preceding self-initiated movement, has been suggested to reflect neural processes underlying the preparation of voluntary action; yet more than fifty years after its introduction, interpretation of the RP remains controversial. Based on previous research showing that internal bodily signals affect sensory processing and ongoing neural activity, we here investigated the potential role of interoceptive signals in voluntary action and the RP. We report that (1) participants initiate voluntary actions more frequently during expiration, (2) this respiration-action coupling is absent during externally triggered actions, and (3) the RP amplitude is modulated depending on the respiratory phase. Our findings demonstrate that voluntary action is coupled with the respiratory system and further suggest that the RP is associated with fluctuations of ongoing neural activity that are driven by the involuntary and cyclic motor act of breathing.

## Abstract (web summary)

Voluntary action and free will have been associated with cortical activity, referred to as “the readiness potential” that precedes self-initiated actions by about 1 s. Here, the authors show that the involuntary and cyclic motor act of breathing is coupled with voluntary action and the readiness potential.

## Introduction

Voluntary action control, the ability to initiate an action based on one’s own will, is an essential component of self-consciousness^1,2^. More than 50 years ago, Kornhuber and colleagues reported that a slow negative drift of brain activity, the so-called readiness potential (RP), precedes the onset of voluntary action by ∼ 1 second^3^, a finding which has been replicated and confirmed by diverse electrophysiological methods including single cell recordings in humans^1^ and animals^4^. Together with Libet’s seminal observation that the onset of the RP precedes participants’ conscious intention of a movement^5^, the RP has been interpreted as an unconscious cortical precursor to conscious intention^6^. Recently, this decade-long interpretation has been challenged, with Schurger and colleagues proposing that the RP reflects spontaneous fluctuation of background neuronal activity or noise, rather than a specific neural process underlying the preparation of voluntary action^7–9^.

An important source of continuously changing neural signals—at the cortical, subcortical, and peripheral level—is interoceptive signals, for example respiratory or cardiac signals. In the present study, we tested whether these interoceptive bodily signals impact voluntary action and the RP. This investigation was motivated by three lines of previous evidence. First, recent research has shown that interoceptive signals and associated neural activity impact sensory processes, including visual perception^10^ and emotional processing^11^, as well as involuntary movements such as micro-saccadic eye movements^12^. Second, previous work suggested that interoceptive neural processing contributes to ongoing neuronal activity at rest,^13–16^ which has been suggested to be associated with RP generation^8,9^. Third, breathing control has been shown to be associated with cortical motor regions such as the supplementary motor area (SMA)^17^, which also has been proposed as the primary source of the RP^1^. In addition, breathing is synchronized with locomotion in mammals^18^ and whisking in rodents^19^, collectively suggesting a potential interaction between breathing and the control of voluntary action. Thus, in the present study, although we measured both heartbeat and breathing signals, we were more interested in the potential link between respiration and voluntary action. In humans, although few studies have measured respiration signals during voluntary movement tasks, it has rather been considered as physiological noise^20^. Based on these three lines of evidence, we predicted that interoceptive signals, in particular respiration, could be the source of the apparently spontaneous fluctuations of background neuronal activity that has been linked to the RP^8^.

To test this hypothesis, we asked participants to perform two classical voluntary action tasks, the Kornhuber task^3,21^ and the Libet task^5^, while recording their electroencephalographic (EEG) and electrocardiographic (ECG) signals, as well as respiration data. Because it has been shown that cardiac phase (e.g., systole vs. diastole)^11,22^ and respiratory phase (e.g., inspiration vs. expiration)^23,24^ affect a range of sensory processes, we focused our analysis on the coupling between voluntary action and the phase of cardiac and respiratory signals. Here, we show that fluctuations of respiratory, but not cardiac, phase are coupled with the onset of voluntary action and the neural hallmark of voluntary action, the RP, and further that such respiration-action coupling is absent during externally triggered actions.

## Results

### Respiratory phase is coupled with voluntary action

**Fig. 1:**
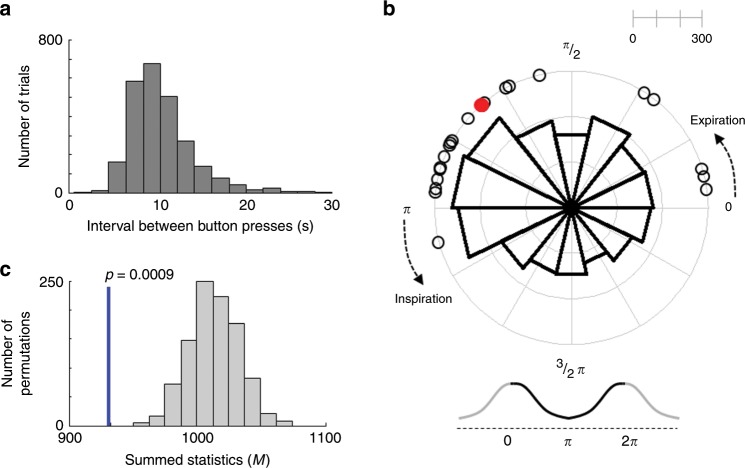
Coupling between voluntary action and respiratory phase during the Kornhuber task in Experiment 1. **a** Distribution of intervals between button presses (pooled across 20 participants) showed the typical response distribution. **b** Distribution of respiration phases with respect to the timing of voluntary action onset. Empty black circles represent each participant’s mean respiration phase at button press. Participants initiated voluntary actions more frequently during the late phase of expiration. The polar histogram shows the distribution of all the button presses from 20 participants, which was also concentrated in the late phase of expiration. The red dot indicates the grand-averaged respiration phase at button press. **c** Histogram of the summed statistics (i.e., minimum number of data points that can be observed in half of the circle; Hodges–Ajne test) obtained from surrogate respiration data. The blue vertical line indicates the summed statistics from original respiration data. The computed test statistics using original data was significantly smaller than chance-level statistics obtained from phase shifted surrogate respiration data (permutation *p* = 0.0009), confirming the timing of button presses in Experiment 1 is coupled with respiratory phase.

In Experiment 1, participants performed the Kornhuber task^3^ and were instructed to press a button on a keypad in a self-initiated manner roughly every 8–12 s. We explicitly instructed participants not to use any strategies such as counting numbers (e.g., seconds) and to try to use irregular intervals to maximize spontaneity of the task, as typically done in this voluntary action task^3,21^. When pooled across 20 participants, the distribution of intervals between button presses (i.e., waiting time) showed a rightward skewed shape (Fig. 1a) in-line with previous voluntary action studies^1,9^. The interval between button presses was on average 11.20 ± 2.30 s (mean ± SD), and the standard deviation of these intervals was on average 3.26 ± 1.70 s, indicating that participants successfully performed the Kornhuber task.

We first tested our hypothesis whether the onset of voluntary movement is associated with spontaneous breathing signals. For this, we computed the phase of respiration at the onset of button presses, using the Hilbert transform (Supplementary Fig. 1). We found that participants pressed the button more frequently during the expiration phase, in particular during the latest phase of expiration, just prior to inspiration onset (Fig. 1b, c; permutation test, *p* = 0.0009). The mean respiration phase at the moment of button press was observed for 19 out of 20 participants during the expiration phase, that is between 0 and π (see open black circles in Fig. 1b). After the experiment was terminated, we asked all participants whether they had been aware of any relationship between their breathing or heartbeat and the button presses (Q1), and whether they had used their breathing or heartbeat to press the button (Q2). Among 20 participants, 18 responded “No” to Q1/Q2, suggesting that our group of participants was unaware of the observed link between voluntary movement and respiration.

**Fig. 2:**
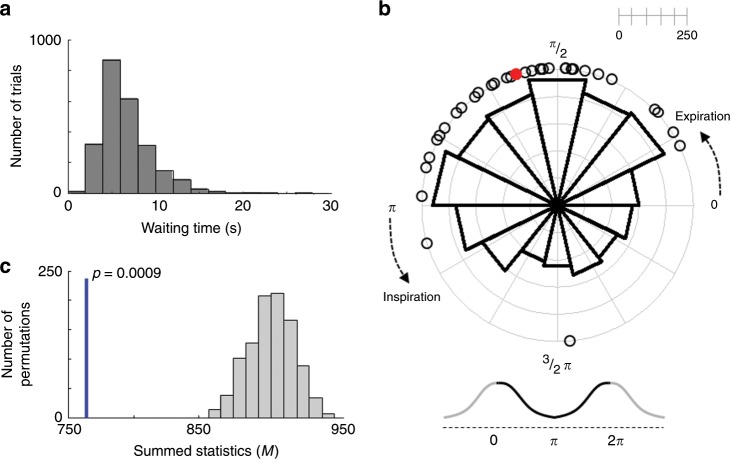
Coupling between voluntary action and respiratory phase during the Libet task in Experiment 2. **a** Distribution of waiting times (pooled across 32 participants) showed the typical rightward skewed shape. **b** Distribution of respiration phases with respect to the timing of voluntary action onset. Empty black circles represent each participant’s mean respiration phase at button press. Participants initiated voluntary actions more frequently during the expiration phase. The polar histogram shows the distribution of all the button presses from 32 participants, which was also concentrated in the expiration phase. The red dot indicates the grand-averaged respiration phase at button press. **c** The computed test statistics using original data (indicated by the blue vertical line) was significantly smaller than chance-level statistics obtained from phase shifted surrogate respiration data (indicated by the histogram; permutation *p* = 0.0009), showing the timing of button presses in Experiment 2 is coupled with respiration phase.

In two further experiments, we aimed to (1) replicate the breathing-voluntary action coupling in a separate group of new participants (*N* = 32) using a different voluntary action task, the classical Libet task^5^ (Experiment 2), and to (2) test whether the observed coupling effect is specific to voluntary action or whether it extends to non-voluntary action (Experiment 3). During the Libet task (Experiment 2), participants were asked to press the button at any time they wanted to, while they were asked to look at a clock face with a rotating red dot. Participants estimated the time when they first felt the intention to press the button (i.e., W-time). The waiting time for the button press was on average 6.67 ± 1.52 s (Fig. 2a), and the standard deviation of waiting time was on average 2.20 ± 0.97 s. W-time was on average −0.26 ± 0.17 s. These behavioral results of waiting time and W-time are comparable with previous studies using the Libet task^1,9^.

Importantly, participants pressed the response button more frequently during the expiration phase (Fig. 2b, c; permutation test, *p* = 0.0009), replicating the results from Experiment 1. The mean respiration phase at the moment of button press was observed for 30 out of the new 32 participants during the expiration phase (see open black circles in Fig. 2b). Upon completion of the study, we asked all participants whether they had been aware of any relationship between their breathing or heartbeat and the button presses (Q1), and whether they had used their breathing or heartbeat to press the button (Q2). From a total of 32 participants, 31 responded “No” to Q1/Q2 (only one participant responded “yes” to (Q1), but not (Q2)).

### Respiratory phase is not coupled with non-voluntary action

During the externally triggered action task (Experiment 3), participants were asked to press the response button as soon as they perceived a green dot at the center of the clock face. The interval from beginning of the clock rotation to the button presses was on average 7.03 ± 1.56 s (Supplementary Fig. 2a) and the standard deviation of intervals was on average 1.98 ± 0.85 s. Permutation test confirmed that during the externally triggered action task, participants’ button presses were not coupled with the breathing phase (Supplementary Fig. 2b, c; permutation test, *p* = 0.32). These results show that the reported coupling between the respiration phase and the movement onset is specifically observed for voluntary actions, but not during externally triggered actions in an otherwise identical experimental setup.

### Cardiac phase is not associated with voluntary action

Next, we checked whether the phase of cardiac signal is associated with voluntary movement onset. For this analysis, the phase of the ECG signal was calculated based on a peak detection algorithm (Supplementary Fig. 3)^25^, rather than the Hilbert transform (see Methods). In Experiment 1, the distribution of cardiac phases at the timing of button press was not non-uniform (Supplementary Fig. 4; Hodges–Ajne test for all the button presses across 20 participants, *p* = 0.80, *M* = 1262; Hodges–Ajne test for mean values across 20 participants, *p* = 0.59, *M* = 6). There was no significant association between the cardiac phase and button press in Experiment 2 (Libet task, Supplementary Fig. 5a; Hodges–Ajne test for all the button presses across 31 participants, *p* = 0.24, *M* = 1106; Hodges–Ajne test for mean values across 31 participants, *p* = 0.71, *M* = 11) nor in Experiment 3 (externally triggered action task, Supplementary Fig. 5b; Hodges–Ajne test for all the button presses across 31 participants, *p* = 0.78, *M* = 1125; Hodges–Ajne test for mean values across 31 participants, *p* = 0.92, *M* = 12). These results indicate participants’ button presses were not associated with the cardiac phase.

Taken together, our behavioral results show that the spontaneous breathing phase, but not the cardiac phase, is coupled with the onset of voluntary action, as tested in two classical voluntary motor tasks, and this respiration-action coupling is absent during externally trigged actions. The questionnaire data further indicate that our participants were not aware of this coupling between respiration and their voluntary actions.

### Coupling between respiratory phase and RP amplitude

**Fig. 3:**
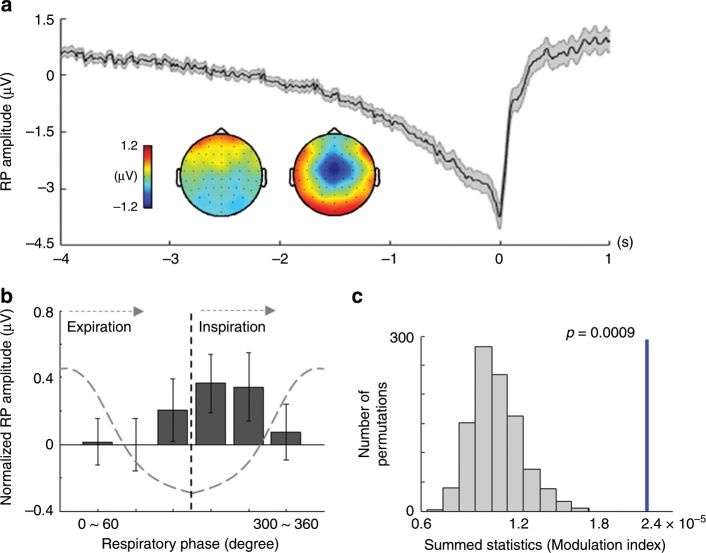
Coupling between the respiratory phase and the readiness potential (RP) amplitude. **a** RP waveform obtained from fronto-central electrodes (*n* = 52). Gray shaded area represents the s.e.m. Inserted topographies were respectively obtained from the time windows (−4 ∼ −2 s; left) and (−2 ∼ 0 s; right). **b** The amplitude of RP as a function of six equally sized bins of the respiratory phase is shown. Error bars represent the s.e.m. **c** Histogram of the summed statistics (i.e., Modulation index; MI) obtained from surrogate respiration data whose phase is randomly shifted. The blue vertical line indicates the summed statistics from original respiration data. The result of the permutation test confirms that the original MI is significantly larger than chance-level MIs obtained from surrogate respiration data (permutation *p* = 0.0009).

Next, we tested the idea that respiration signals could affect the RP, based on the observed behavioral coupling between respiration and voluntary action. For this, we combined EEG-respiration data from Experiment 1 and Experiment 2, because (1) these two classical paradigms (Kornhuber task, Libet task) have been most commonly used in RP research, because (2) we observed similar breathing-action coupling in both voluntary action tasks, and because (3) we thereby wanted to increase statistical power for single trial RP analysis^26^. We first identified typical RPs by averaging EEG signals preceding the onset of voluntary finger movements (Fig. 3a). In accordance with previous findings, RPs were observed in central and fronto-central regions beginning ∼2 s before the voluntary action onset.

We next tested directly whether the RP amplitude is associated with the phase of concurrently measured respiration signals. In brief, we computed the mean RP amplitude in each single trial depending on six equally sized bins of the respiration phase. Then, to determine the statistical significance of coupling between respiration phase and RP amplitude, a Modulation index (MI) was computed in each participant (see Methods)^15,27,28^. The results show that the RP amplitude was smaller during the expiration periods compared with the inspiration periods (see Fig. 3b). Permutation test confirmed the significant phase-amplitude coupling between respiration and RP by showing that the MI computed using the original data is significantly larger than surrogate MIs obtained from phase shifted respiration signals (Fig. 3c; permutation *p* = 0.0009).

### Resting state EEG is not modulated by respiratory phase

**Fig. 4:**
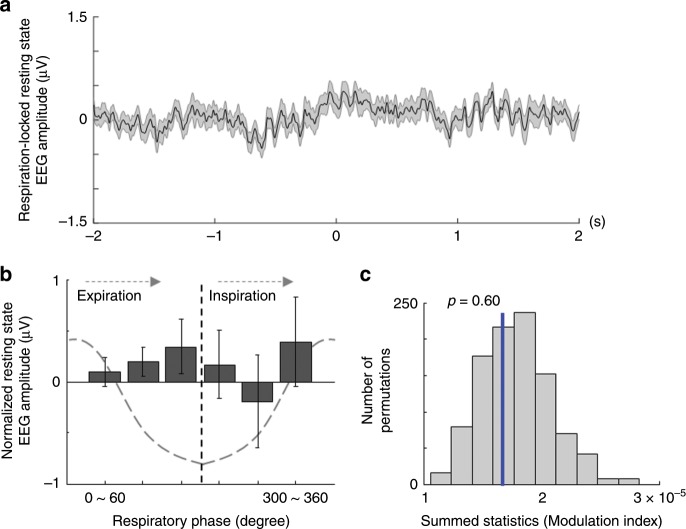
Respiratory phase and resting state EEG amplitude. **a** Waveform of resting state EEG amplitude time-locked to the respiration (i.e., inspiration) peak obtained from fronto-central electrodes (*n* = 50). Gray shaded area represents the s.e.m. **b** Resting state EEG amplitude as a function of six equally sized bins of respiratory phase. Error bars represent the s.e.m. **c** Histogram of the summed statistics (i.e., Modulation index) obtained from surrogate respiration data whose phase is randomly shifted. The blue vertical line indicates the summed statistics from original respiration data. The result suggests that respiration-locked resting state EEG amplitude does not depend on the respiratory phase (permutation *p* = 0.60).

In a final EEG analysis, we investigated whether the phase of respiration is related to the modulation of resting state EEG amplitude, to exclude the possibility that the observed coupling between the RP amplitude and respiration phase might reflect mere artefactual influence of respiration on EEG signals. For that we computed the resting state EEG amplitude that was time-locked to the respiration peak (i.e., inspiration), from the same electrodes that were used for RP computation, and then computed the MI between the resting state EEG amplitude and the respiration phase (Fig. 4a, b). Analysis (permutation test) showed that the computed MI using the original data are not different from the chance-level MIs obtained from phase shifted respiration data (Fig. 4c; *p* = 0.60). This shows that the resting state EEG amplitude time-locked to the respiration peak does not depend on the respiration phase, indicating that the coupling between respiration signals and the RP amplitude is not related to artefactual influence of the respiration signal on EEG activity at rest.

## Discussion

The first major observation of the present study is that the breathing pattern of our participants was systematically coupled with the onset of their voluntary movements, despite the fact that our participants were entirely free to choose the movement onset within the experimental constraints. These results were obtained using the two most frequently used voluntary action paradigms: the Kornhuber (Experiment 1) and the Libet task (Experiment 2) and were obtained in two different subject samples. It has been pointed out that, whereas the Kornhuber paradigm may involve additional cognitive task-related components such as interval estimation, this is not the case during the Libet task^9^, confirming the specific coupling between respiration and voluntary action. In addition, we showed that respiration phase was not coupled with involuntary action (i.e., externally triggered action) in Experiment 3. Taken together, our findings from three experiments show that respiration phase is associated with voluntary action, but not interval estimation or externally triggered actions.

Whereas previous work has demonstrated that interoceptive processing (including respiration) influences diverse sensory processes^10,11,24^, the present findings provide experimental evidence that interoceptive processing is associated with voluntary action control in humans. Breathing is inevitably linked to orofacial movements such as speaking^29^ and swallowing^30^, owing to their shared anatomical structures (e.g., the pharynx and larynx) for airflow control. Animal electrophysiological studies have also investigated such coupling between breathing and lower-level motor functions such as locomotion in mammals and whisking in rodents^18,19^, showing, for example, that whisking is phase-locked to sniffing (i.e., fast breathing pattern whose rates are higher than 5 Hz) during active exploration^19^. Of note, such motor-respiratory coupling is mostly linked to involuntary movements, automatic respiratory-motor control, and the maintenance of the metabolic homeostasis. The present results showing the relationship between breathing and voluntary action control goes beyond these homeostatic controls and reflexes, revealing that the respiratory system affects voluntary action, a higher-level motor control function that has been associated with free will and self-consciousness in humans^2^.

We further observed that the amplitude of the RP, which is the neural hallmark of voluntary action, is coupled with the phase of simultaneously recorded respiration signals. We computed the MI between the respiration phase and the RP amplitude, following previous studies measuring phase-amplitude coupling between two electrophysiological signals^15,27,28^, and observed smaller RP amplitude during the expiration compared with the inspiration period. We argue that this finding is in accordance with the recent proposal that the RP reflects fluctuations of background neuronal activity^4,8,9^, rather than specific neural events associated with the motor preparation for the button press. Applying evidence accumulation modeling to the voluntary action paradigm, recent work showed that the timing of voluntary action and the associated RP can be explained by the accumulation of stochastic fluctuations in neural activity, which eventually reaches a decision threshold^4,9^, and it has been suggested that the RP might reflect “ebb and flow of background neuronal noise”^8^. The present data are of relevance for the origin of such stochastic neuronal fluctuations that eventually generate the RP. Interestingly, Murakami and colleagues^4^ proposed that potential sources of such variability in neuronal fluctuations could be contributions from multiple body maps including somatosensory, proprioceptive, and interoceptive systems. In accordance with this proposal and based on the present data we argue that such fluctuations of background neuronal activity are neither “noise” nor “spontaneous”, as they are, at least partly, accounted for by respiration signals. This interpretation is further strengthened by recent findings that interoceptive processing, in particular respiratory signal processing, is an important source of “spontaneous” ongoing neural activity^15,16^. Taken together, we propose that the neuronal fluctuations preceding the onset of voluntary action are not (only) spontaneous noise, accumulating based on RP-averaging procedures, but also related to a person’s breathing cycle that modulates the voluntary movement and the related brain activity. In brief, our findings support recent proposals that the RP reflects fluctuations of ongoing neural activity, but further show that the mostly involuntary and cyclic motor act of breathing is an important source of such fluctuations.

The present study also raises new questions about the neural mechanisms of coupling between breathing and voluntary action. We observed that participants initiated voluntary actions more frequently during the expiration period, and least frequently during the inspiration period. Although speculative, we propose that the observed coupling between respiration and voluntary action may occur to reduce the potential competition between two motor commands (i.e., respiration-related motor command vs voluntary motor signal for the finger movement) at the cortical (e.g., SMA)^1^ or subcortical level (e.g., parabrachial nucleus)^29^. In other words, considering that a single breathing cycle is initiated by inspiration and terminated by expiration, participants might have unconsciously preferred to press the button at the end of a single breathing cycle or in between two consecutive breathings. Relatedly, concerning our EEG data, phase-amplitude coupling was not observed between respiration phase and resting state EEG amplitude, in accordance with previous research reporting that respiration-locked EEG modulations were not observed in central regions (e.g., Cz) when participants are breathing quietly at rest^31^. This finding confirms that the coupling between respiration signals and the RP amplitude is not related to artefactual influence of the respiration signal on EEG activity at rest^16^. It further suggests such coupling might involve neural interactions between the premotor areas, which are proposed as neural sources of the RP^1^, and other cortical (e.g., the insula, cingulate cortex) or subcortical regions (e.g., ventrolateral medulla), which are known to be associated with spontaneous breathing control^32^. Future EEG-fMRI studies as well as invasive recordings in humans will also be helpful to investigate these issues and help delineate the neural structures and mechanisms underlying the present effects. We note, that breathing signals are ideally suited among other interoceptive inputs, to test coupling between interoception and voluntary action. They are more easily rendered conscious and more easily accessible for experimental manipulations, such as loaded breathing that has been shown to modulate breathing parameters, emotional processing, and self-consciousness^33,34^. Breathing is also intimately linked to a large range of motor activities, as well as cognitive activities such as language^35^. Breathing awareness is another modulating factor in breathing control in health and disease^36^. Interestingly, recent research showed that nasal, but not oral, respiration induces cortical oscillations, and affects associated cognitive processes^15,24^, suggesting that different respiration-related mechanisms (e.g., nasal vs. oral) or individual aspects (e.g., athletes vs. non-athletes) may modulate the RP. Future research will help in detailing the subjective, behavioral, physiological, and neural mechanisms involved in the coupling between breathing control, voluntary action, and other cognitive functions.

In conclusion, we here show that spontaneous breathing impacts a fundamental aspect of human self-consciousness and motor cognition, namely voluntary action, as well as one of the most classical EEG components, the RP. Our findings provide new insights into the RP by joining two previously disparate fields of neuroscience proposing that (1) the RP is associated with fluctuations of ongoing neuronal activity^4,7–9^, and that (2) interoceptive processing, in particular processing related to respiration, is an important source of such ongoing neuronal fluctuations^15^. Bridging the gap between these two separate fields we show that the RP during self-initiated movements is indeed associated with the fluctuations of ongoing neuronal activity that are driven by the respiratory system. Finally, our results might also be of relevance to resolve the puzzling questions that were initially raised by Benjamin Libet between the timing of the RP and related neural activity with respect to the subjective sensation or intention to move^5^. We propose that the RP does not correspond to the “unconscious cerebral initiation of a voluntary action”^6^, but at least partly reflects respiration-related cortical processing that is coupled to the onset of voluntary action.

## Methods

### Participants

In Experiment 1, 20 participants (10 female; 20 right-handed; mean age: 26 ± 1.3 years) took part in the study. In Experiments 2 and 3, 34 participants (15 female; 31 right-handed; mean age: 26.5 ± 5.1 years) conducted the experiments. Two participants were excluded from analysis owing to the excessive movement artifacts contaminating > 50% of both respiration and EEG signals. In addition, one participant was excluded from cardiac phase analysis owing to noisy ECG signals, and two participants were excluded from resting state data analysis owing to missing trigger signals. All participants reported no history of neurological or psychiatric disorders nor cardiovascular diseases. All subjects signed a written informed consent form and were paid for their participation. All procedures were approved by the local ethics committee (Commission Cantonale d’Ethique de Genève).

### Paradigm

In Experiment 1, participants performed the Kornhuber task^3,21^. An experimental session consisted of three blocks of 8 min. Participants were instructed to press a button on a keypad voluntarily using their right index finger. To produce one voluntary movement per roughly three respiration cycles, participants were asked to press the button every 8–12 s. Importantly, participants were asked (1) not to count any numbers (e.g., seconds) to estimate the time, (2) to avoid making regular or rhythmic button presses^3,9^ to maximize the spontaneity of the task. Before the real recordings, participants conducted a short training session (∼1 min) and the experimenter gave feedback on the interval and regularity of their button presses, so that participants could adjust them. Throughout the experiment, participants were acoustically isolated with continuous white noise via insert earphones (MX 365, Sennheiser), and closed eyes. We excluded the first trials in each block from the analysis (<3%), as participants often involuntarily pressed the key at the beginning of white noise. Inclusion of such trials did not affect the results. At the end of Experiment 1, resting state EEG data (for 3 min) were acquired while participants had their eyes closed.

An independent group of participants conducted Experiments 2 and 3. In Experiment 2, participants performed the Libet task^5^. A trial was initiated when a red dot appeared at a random location of the clock face (radius: 2˚ of visual angle). The red dot then rotated at 2560 ms per cycle. Participants were instructed to wait for at least one full rotation and after that to press the button at any time they wanted by using their right index finger. In-line with previous studies, we asked participants to avoid (1) pre-planning the location of the dot for the button press, and (2) using same intervals across trials (for previous work and instructions see refs. ^5,9,37^). After the participant pressed the button, the red dot disappeared immediately. After 4 s, the red dot reappeared at the same location, and the participants indicated the clock position which they first felt the conscious urge (or intention) to press the button using the keypad. After a random inter-trial interval (i.e., 4–8 s), the next trial began.

In the externally triggered task of Experiment 3, participants were asked to press the button as fast and accurately as possible after detecting a green dot, which appeared for 200 ms at the fixation point, while the red dot was rotating (i.e., as in the Libet task). Once the button was pressed the red dot disappeared immediately. After a random inter-trial interval (i.e., 4–8 s), the next trial began. The interval of the green dot appearance was based on each participant’s performance during the previous Libet experiment. Participants performed three blocks (i.e., 75 trials in total; one participant conducted 90 trials) for Experiments 2 and 3. In order to collect the individual interval data for the green dot appearance in Experiment 3, the experiment always began with the Libet task, whereas the remaining five blocks were pseudo randomized. After participants had completed Experiments 2 and 3, we acquired EEG resting state data for 5 min, while participants were asked to fixate the center of the screen.

### Respiration recording and analysis

Continuous respiration signal was collected using a respiration belt (Biopac MP36, Biopac System Inc) at a sampling rate of 2000 Hz. Preprocessing and averaging were conducted using the Fieldtrip toolbox^38^. To determine the instantaneous respiration phase at the timing of button presses, we first bandpass filtered the continuous respiration signal between 0.2 and 0.8 Hz, and then applied the Hilbert transform (Supplementary Fig. 1). We detected inspiration peaks by correlating respiration signal with a template defined on a subject-by-subject basis^10^. For the MI analysis, continuous respiration data were down sampled to 512 Hz.

### EEG recording and analyses

EEG signals were collected using a 64-channel active electrode EEG system (ActiveTwo system, Biosemi) at a sampling rate of 2048 Hz and online low-pass filtered at 400 Hz. Continuous EEG data were down sampled to 512 Hz and offline filtered between 0.1 and 40 Hz, following a recent observation that applying a high pass filtering at 0.1 Hz effectively reduces infra slow oscillations when computing slow brain potentials such as the RP^39^. EEG data were re-referenced to a common average reference, as in a recent RP study^40^.

RP was computed on EEG signals locked to the onset of the button press. After epoching (−4 to 1 s regarding the movement onset), trials showing excessive noise (i.e., > 3 SD) were excluded from further analysis. After artifact correction, 118 ± 24 (in Experiment 1) and 68 ± 4 epochs (in Experiment 2) were averaged in each subject to compute the RP. Baseline correction was not applied^9^. RPs are typically observed in fronto-central electrodes^3,7,9,40^. To maximize the signal-to-noise ratio, we report the RP results from electrodes that showed the highest RP amplitude among fronto-central electrodes (i.e., Cz, FCz, Fz, AFz), defined on a subject-by-subject basis.

### ECG recording and analysis

ECG signals were simultaneously recorded using the above-mentioned EEG amplifier and also the same preprocessing was applied to both EEG and ECG signals as described above. Bipolar ECG electrodes were placed over the right shoulder and the bottom of the left side of the abdomen. To compute the phase of ECG signal at the button press, we applied a method based on a peak detection algorithm, as the Hilbert transform cannot be applied to the ECG signal which does not have oscillatory shape. For that, R-peaks were detected by correlating the ECG signal with a template QRS complex defined on a subject-by-subject basis^10^. Then the phase of the ECG signal was calculated (Supplementary Fig. 3) using the following formula: *φ*(t) = 2π((t – ta)/(tb − ta)), where ta and tb are the timings of two successive peaks surrounding the current time sample^25^.

### Statistical test of breathing-voluntary action coupling

The significance of the relationship between the timing of button presses and the phase of respiration signals was tested using a permutation-based two-step process. For each participants, we first applied the Hodges–Ajne test (or Omnibus test), which assesses the uniformity of circular data such as a phase distribution without assumptions on the distribution of the data^41^, as implemented in the Circular Statistics Toolbox^42^. The Hodges–Ajne test results in a test statistic (i.e., *M*) defined as the minimum number of data points that can be observed in half of the circle. The null hypothesis of uniform distribution is rejected when the test statistic is smaller than the expected numbers^41^. Importantly, considering that the expiration duration is longer than inspiration one (see Supplementary Fig. 1), it is expected that even completely random events that are not associated with breathing cycle will be more likely observed during the expiration phase. Thus, as a second step, the computed original statistic (i.e., sum of *M* across all participants) was compared with the null distribution of surrogate *M* values that are obtained from phase shifted respiration data. For that the phase of respiration signals was cut into two segments with a random amount, and the order was swapped in each block and subjects^27^. We created 1000 surrogate *M* values that define the chance-level coupling between the respiration phase and button presses. Then, a two-sided permutation *p* value was obtained.

### MI between RP amplitude and respiratory phase

For assessing the coupling between the respiratory phase and the RP amplitude, we first computed the RP amplitude and respiration phase in each single trial in a −4 to 0 s time-window (i.e., around one respiratory cycle) regarding the onset of the button press. Then, the RP amplitude was averaged depending on six equally sized respiration phase bins that spanned the 0–2π interval. Trials that did not result in six mean RP amplitudes across six respiration phase bins (<2% of total trials) were excluded from further analysis. Sorted RP amplitudes in each breathing phase bins were normalized (i.e., dividing by the sum of all RP amplitudes in each trial; see Fig. 3). The degree of coupling between the sorted RP amplitude and respiration phase was quantified by computing the MI^15,28^, which quantifies how much a given distribution of amplitudes across phase bins deviates from a uniform distribution, using mean RP amplitudes across single trials in each six respiration phase bin for each subject. Stronger phase-amplitude coupling (i.e., more deviation from uniform distribution) results in the higher MI values. MI significance was tested using a permutation test for which we first computed grand-averaged MI across all participants, using the original EEG-respiration data. Then the original grand-averaged MI was compared with the null distribution of surrogate MI values whose phase-amplitude association was disrupted by randomly shifting the respiration phase data as explained above. We created 1000 surrogate MI values which define the chance-level coupling between the RP amplitude and respiration phase. Finally, a two-sided permutation *p* value was obtained.

To compute the MI between respiration phase and resting state EEG amplitude, EEG data were epoched in −2 to 2 s time windows around the inspiration peak (see Fig. 4a). The same procedure was then applied between the resting state EEG amplitude and the concurrently measured respiration phase, as explained above.

### Reporting summary

Further information on research design is available in the Nature Research Reporting Summary linked to this article.

## Supplementary information

Supplementary Information

Reporting Summary

## Data Availability

The data that support the findings of this study are available from the corresponding authors upon reasonable request.

## Code availability

All code is available from the corresponding authors upon reasonable request.
