# Supplementary material for: Breathing is coupled with voluntary action and the cortical readiness potential
Source: Nat Commun. 2020 Feb 6;11:289. doi: 10.1038/s41467-019-13967-9 (PMC7005287; doi:10.1038/s41467-019-13967-9)
Supplement: Supplementary file 1 — Supplementary Information [file 41467_2019_13967_MOESM1_ESM.pdf]

Breathing is coupled with voluntary action and the cortical readiness potential

Park et al.

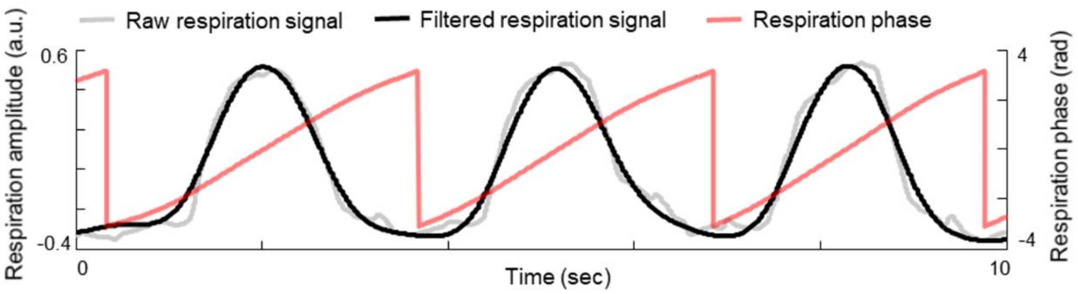

Supplementary Figure 1. Computation of respiration phase. Raw respiration signals were bandpass filtered between 0.2 and 0.8 Hz. Then the respiration phase was obtained using the Hilbert transform.

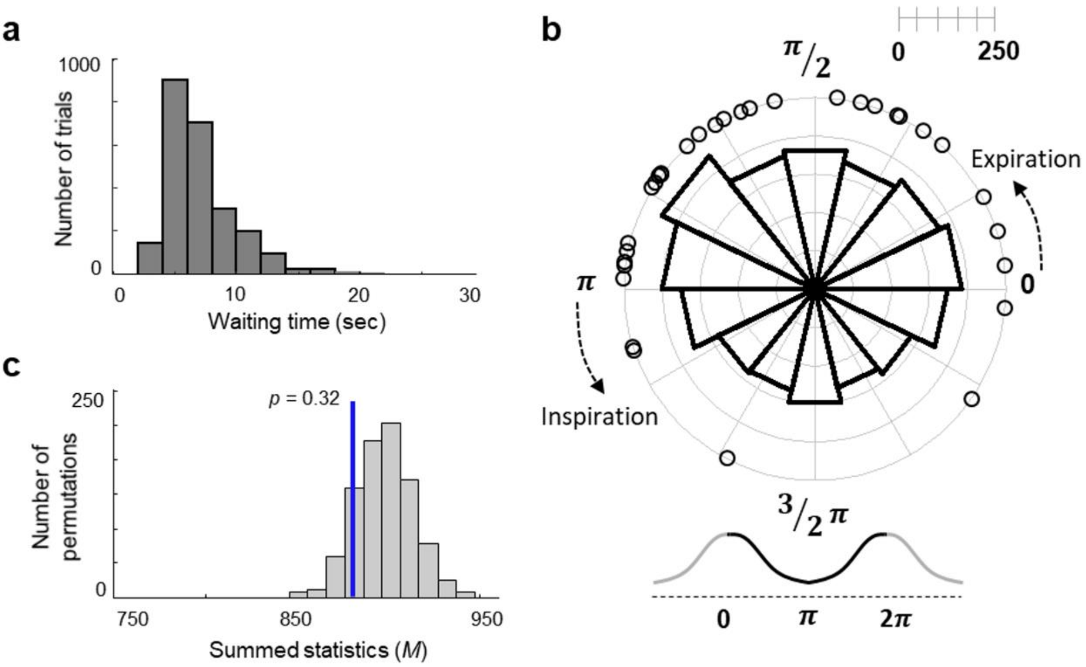

Supplementary Figure 2. The respiratory phase and the onset of externally-triggered button presses in Experiment-3. (a) Distribution of intervals from beginning of trial to the externally-triggered button presses (pooled across 32 participants). (b) Distribution of respiration phases with respect to the timing of externally-triggered button presses. The small black circle represents each participant's mean respiration phase at the button press. The polar histogram shows the distribution of all the externally-triggered button presses from 32 participants. (c) Histogram of the summed statistics (i.e., minimum number of data points that can be observed in half of the circle; Hodges-Ajne test) obtained from surrogate respiration data. The blue vertical line indicates the summed statistics from original respiration data. A computed test statistics using original data was not different from chance level statistics obtained from phase shifted surrogate respiration data (permutation p = 0.32), confirming the timing of button press in Experiment-3 is not coupled with respiration phase.

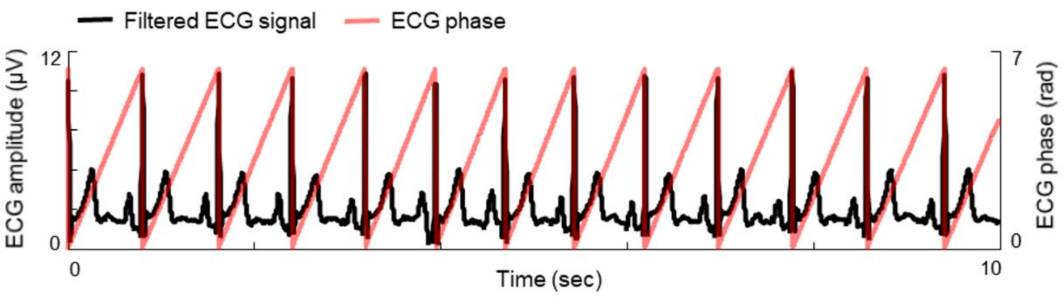

Supplementary Figure 3. Computation of cardiac phase. Heartbeat phase was computed using a method based on a peak detection algorithm.

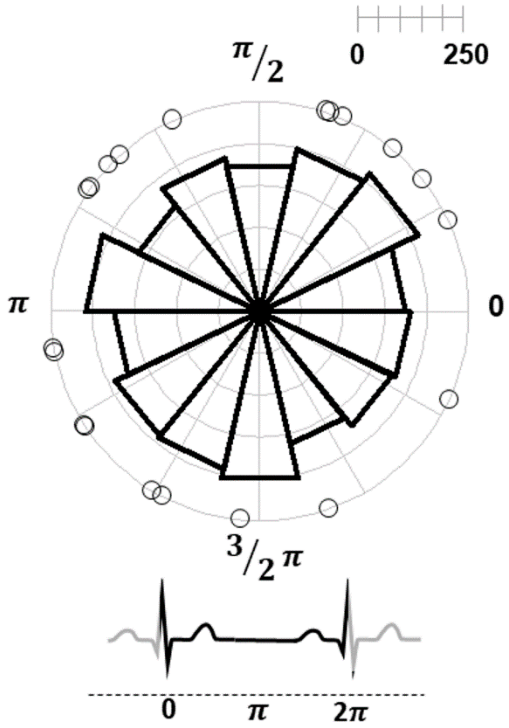

Supplementary Figure 4. Distribution of heartbeat phases at the timing of the voluntary action in Experiment-1. The small black circle represents each participant's mean heartbeat phase at the button press. No preferential phase emerged (Hodges-Ajne test, p = 0.59, M = 6). Polar histogram shows the distribution of all the button presses from 20 participants, which was uniformly distributed (p = 0.80, M = 1262).

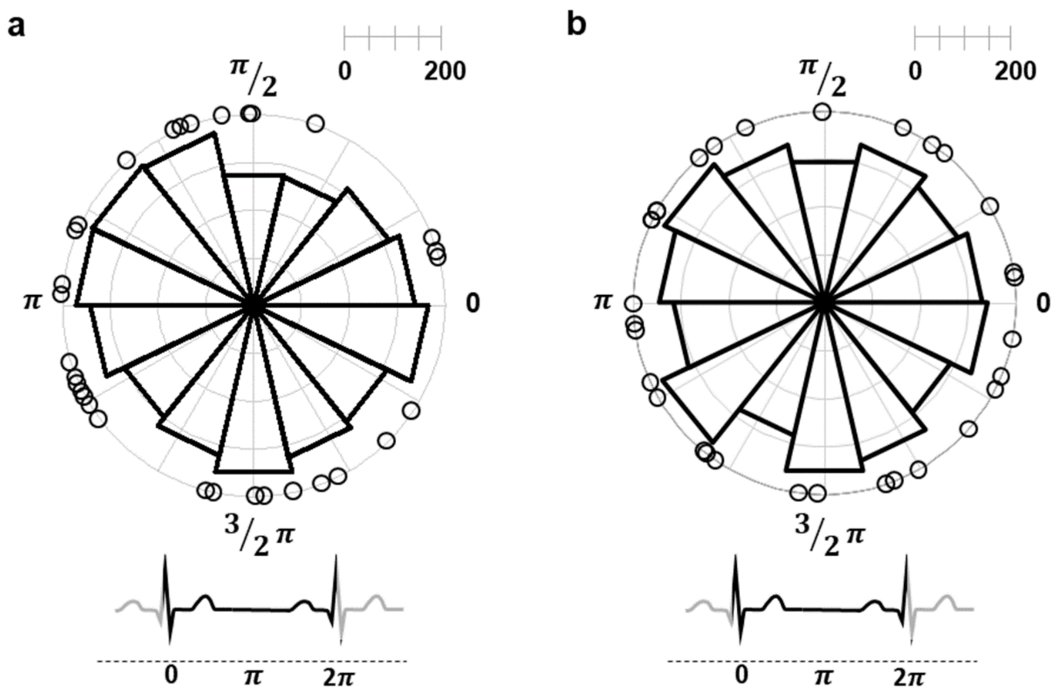

Supplementary Figure 5. Distribution of heartbeat phases at the timing of the button presses in Experiment-2 and 3. The small black circle represents each participant's mean heartbeat phase at the button press. No preferential phase emerged neither in the Libet task (a: Hodges-Ajne test, p = 0.71, M = 11) nor in the externally-triggered task (b: p = 0.92, M = 12). Polar histogram shows the distribution of all the button presses from 31 participants, which was uniformly distributed (a: p = 0.24, M = 1106; b: p = 0.78, M = 1125).
