# Supplementary material for: Breathing is coupled with voluntary action and the cortical readiness potential
Source: Nat Commun. 2020 Feb 6;11:289. doi: 10.1038/s41467-019-13967-9 (PMC7005287; doi:10.1038/s41467-019-13967-9)
Supplement: Supplementary file 2 — Reporting Summary [file 41467_2019_13967_MOESM2_ESM.pdf]

nature research

Corresponding author(s): Hyeong-Dong Park; Olaf Blanke

Last updated by author(s): Dec 10, 2019

Reporting Summary

Nature Research wishes to improve the reproducibility of the work that we publish. This form provides structure for consistency and transparency in reporting. For further information on Nature Research policies, see Authors & Referees and the Editorial Policy Checklist.

Statistics

For all statistical analyses, confirm that the following items are present in the figure legend, table legend, main text, or Methods section.

n/a	Confirmed	
☐	☒	The exact sample size (n) for each experimental group/condition, given as a discrete number and unit of measurement
☐	☒	A statement on whether measurements were taken from distinct samples or whether the same sample was measured repeatedly
☐	☒	The statistical test(s) used AND whether they are one- or two-sided<br>*Only common tests should be described solely by name; describe more complex techniques in the Methods section.*
☒	☐	A description of all covariates tested
☐	☒	A description of any assumptions or corrections, such as tests of normality and adjustment for multiple comparisons
☐	☒	A full description of the statistical parameters including central tendency (e.g. means) or other basic estimates (e.g. regression coefficient) AND variation (e.g. standard deviation) or associated estimates of uncertainty (e.g. confidence intervals)
☐	☒	For null hypothesis testing, the test statistic (e.g. F, t, r) with confidence intervals, effect sizes, degrees of freedom and P value noted<br>*Give P values as exact values whenever suitable.*
☒	☐	For Bayesian analysis, information on the choice of priors and Markov chain Monte Carlo settings
☒	☐	For hierarchical and complex designs, identification of the appropriate level for tests and full reporting of outcomes
☐	☒	Estimates of effect sizes (e.g. Cohen's d, Pearson's r), indicating how they were calculated

Our web collection on statistics for biologists contains articles on many of the points above.

Software and code

Policy information about availability of computer code

Data collection	ActiView (BioSemi) for EEG recording; AcqKnowledge (Biopac) for respiration recording
Data analysis	Fieldtrip toolbox (ver. 20150907); CircStat toolbox (ver. 2012)

For manuscripts utilizing custom algorithms or software that are central to the research but not yet described in published literature, software must be made available to editors/reviewers. We strongly encourage code deposition in a community repository (e.g. GitHub). See the Nature Research guidelines for submitting code & software for further information.

Data

Policy information about availability of data

All manuscripts must include a data availability statement. This statement should provide the following information, where applicable:

- Accession codes, unique identifiers, or web links for publicly available datasets
- A list of figures that have associated raw data
- A description of any restrictions on data availability

A reporting summary for this Article is available as a Supplementary Information file. The data that support the findings of this study are available from the corresponding authors upon reasonable request.

Field-specific reporting

Please select the one below that is the best fit for your research. If you are not sure, read the appropriate sections before making your selection.

☐ Life sciences ☒ Behavioural & social sciences ☐ Ecological, evolutionary & environmental sciences

Behavioural & social sciences study design

All studies must disclose on these points even when the disclosure is negative.

Study description	Quantitative experimental study
Research sample	20 participants (10 female; 20 right-handed; mean age: 26 ± 1.3 years; villagers in regional area) in Experiment-1. 34 participants (15 female; 31 right-handed; mean age: 26.5 ± 5.1 years; villagers in regional area) in Experiment-2 and 3.
Sampling strategy	The sampling procedure was random. No statistical test was run to determine sample size a priori. The sample sizes we chose are similar to those used in previous publications.
Data collection	A 64-channel active electrode EEG system (ActiveTwo system; Biosemi) and a respiration recording system (Biopac MP36, Biopac System Inc, Canada) were used respectively for recording EEG and respiration data. We were blind to the experimental conditions, and the data collection / analyses were automated to avoid the introduction of bias.
Timing	Start date: 2018-04-11<br>Stop date: 2019-06-14
Data exclusions	Two participants were excluded from analysis due to the excessive movement artifacts contaminating more than 50% of both respiration and EEG signals. In addition, 1 participant was excluded from cardiac phase analysis due to noisy ECG signals, and 2 participants were excluded from resting state data analysis due to missing trigger signals.
Non-participation	No participant declined participation.
Randomization	Participants were not allocated into experimental groups.

Reporting for specific materials, systems and methods

We require information from authors about some types of materials, experimental systems and methods used in many studies. Here, indicate whether each material, system or method listed is relevant to your study. If you are not sure if a list item applies to your research, read the appropriate section before selecting a response.

Materials & experimental systems

n/a	Involved in the study	
☒	☐	Antibodies
☒	☐	Eukaryotic cell lines
☒	☐	Palaeontology
☒	☐	Animals and other organisms
☐	☒	Human research participants
☒	☐	Clinical data

Methods

n/a	Involved in the study	
☒	☐	ChIP-seq
☒	☐	Flow cytometry
☒	☐	MRI-based neuroimaging

Human research participants

Policy information about studies involving human research participants

Population characteristics	Participants reported no history of neurological or psychiatric disorders nor cardiovascular diseases.
Recruitment	Participants were recruited through a online recruiting system (https://epflunil.sona-systems.com/Default.aspx?ReturnUrl=%2f).
Ethics oversight	All procedures were approved by the local ethics committee (Commission Cantonale d'Ethique de Genève).

Note that full information on the approval of the study protocol must also be provided in the manuscript.
